# Chondroitin Sulphate Proteoglycan Axonal Coats in the Human Mediodorsal Thalamic Nucleus

**DOI:** 10.3389/fnint.2022.934764

**Published:** 2022-07-06

**Authors:** Harry Pantazopoulos, Nayeem Mubarak Hossain, Gabriele Chelini, Peter Durning, Helen Barbas, Basilis Zikopoulos, Sabina Berretta

**Affiliations:** ^1^Department of Psychiatry and Program in Neuroscience, University of Mississippi Medical Center, Jackson, MS, United States; ^2^Department of Health Sciences, Boston University, Boston, MA, United States; ^3^Translational Neuroscience Laboratory, Mclean Hospital, Belmont, MA, United States; ^4^Department of Psychiatry, Harvard Medical School, Boston, MA, United States; ^5^Department of Anatomy and Neurobiology, Boston University School of Medicine, Boston, MA, United States; ^6^Neural Systems Laboratory, Boston University, Boston, MA, United States; ^7^Program in Neuroscience, Harvard Medical School, Boston, MA, United States

**Keywords:** axonal coat, brevican, NG2, extracellular matrix, thalamus

## Abstract

Mounting evidence supports a key involvement of the chondroitin sulfate proteoglycans (CSPGs) NG2 and brevican (BCAN) in the regulation of axonal functions, including axon guidance, fasciculation, conductance, and myelination. Prior work suggested the possibility that these functions may, at least in part, be carried out by specialized CSPG structures surrounding axons, termed axonal coats. However, their existence remains controversial. We tested the hypothesis that NG2 and BCAN, known to be associated with oligodendrocyte precursor cells, form axonal coats enveloping myelinated axons in the human brain. In tissue blocks containing the mediodorsal thalamic nucleus (MD) from healthy donors (*n* = 5), we used dual immunofluorescence, confocal microscopy, and unbiased stereology to characterize BCAN and NG2 immunoreactive (IR) axonal coats and measure the percentage of myelinated axons associated with them. In a subset of donors (*n* = 3), we used electron microscopy to analyze the spatial relationship between axons and NG2- and BCAN-IR axonal coats within the human MD. Our results show that a substantial percentage (∼64%) of large and medium myelinated axons in the human MD are surrounded by NG2- and BCAN-IR axonal coats. Electron microscopy studies show NG2- and BCAN-IR axonal coats are interleaved with myelin sheets, with larger axons displaying greater association with axonal coats. These findings represent the first characterization of NG2 and BCAN axonal coats in the human brain. The large percentage of axons surrounded by CSPG coats, and the role of CSPGs in axonal guidance, fasciculation, conductance, and myelination suggest that these structures may contribute to several key axonal properties.

## Introduction

In recent years, a large number of studies have demonstrated the importance of the extracellular matrix (ECM) in the regulation of developmental and adult brain processes, including synaptic plasticity and receptor trafficking (Dityatev et al., 2006; Gundelfinger et al., 2010; Frischknecht and Gundelfinger, 2012), neuronal migration (Charvet et al., 1998; Klausmeyer et al., 2011), axon guidance (Snow et al., 2003; Kwok et al., 2012), oligodendrocyte differentiation (Ichihara-Tanaka et al., 2006; Bekku and Oohashi, 2010; Kucharova and Stallcup, 2010), and regulation of nodes of Ranvier (Bekku et al., 2009; Dours-Zimmermann et al., 2009). In addition, ECM dysregulation has been implicated in several brain disorders, including schizophrenia (Pantazopoulos et al., 2010; Consortium, 2014), bipolar disorder (Cichon et al., 2011; McGrath et al., 2013; Steullet et al., 2018), and Alzheimer’s disease (Bruckner et al., 1999; Morawski et al., 2010; Yang et al., 2017).

The brain ECM surrounds all cells, occupying a volume fraction of approximately 20% of the adult brain (Thorne and Nicholson, 2006; Sykova and Nicholson, 2008). It is composed of aggregates of hyaluronan and chondroitin sulfate proteoglycans (CSPGs) connected by glycoproteins (Yamaguchi, 2000; Rauch, 2004). CSPGs consist of core proteins with varying numbers of chondroitin sulfate (CS) glycosaminoglycan chains. The chemical composition and representation of CSPGs are regulated throughout development and adulthood, modulating properties required for regulating cell migration, myelination, and axon growth (Bandtlow and Zimmermann, 2000; Wang et al., 2008; Giger et al., 2010; Maeda, 2010). In addition to a loosely organized lattice, the ECM forms highly organized structures, including pericellular ECM aggregates called perineuronal nets (PNNs), described as early as 1898 (Golgi, 1898; Celio et al., 1998). PNNs form around subsets of neurons typically during critical periods, regulating synaptic plasticity (Pizzorusso et al., 2002; Gogolla et al., 2009; Mauney et al., 2013). These structures have been the primary focus of studies establishing the importance of CSPGs in adult neural functions. However, evidence for the existence of other ECM structures, such as periaxonal aggregates, has also been reported (Bruckner et al., 2008; Morawski et al., 2010; Lendvai et al., 2012). Periaxonal aggregates were first described by Bruckner et al. who named them “axonal coats” (Bruckner et al., 2008; Morawski et al., 2010). These findings were later reconsidered by these authors, who suggested that in the human lateral geniculate nucleus, these structures may instead correspond to perisynaptic aggregates (Lendvai et al., 2012). However, preliminary observations by our group suggested that, at least in the mediodorsal nucleus of the thalamus (MD), CSPGs do form tubular structures surrounding axons. These preliminary findings prompted further investigations.

Indirect but compelling evidence for the presence of axonal coats comes from a growing body of literature supporting the involvement of the ECM in axonal functions. For example, CSPGs regulate myelination, fasciculation, saltatory impulse conduction, and synaptic functions during development and adulthood. During developmental stages, CSPGs are involved in axonal guidance, including cortico-thalamic axons, and changes in CS sulfation have profound effects on this process (Viapiano and Matthews, 2006; Miyata et al., 2012; Berretta et al., 2015; Fawcett, 2015). Furthermore, several CSPGs play key roles in oligodendrocyte maturation, which in turn impacts myelination of cortico-thalamic axons (Sim et al., 2006; Faissner et al., 2010; Takahashi et al., 2011). During development, glia-derived CSPGs have been shown to regulate axon growth, axon guidance, and axonal fasciculation (Wilson and Snow, 2000; Snow et al., 2003; Ichijo, 2004; Klausmeyer et al., 2011). Importantly, during late development, CSPGs are key contributors to the powerful inhibition that CNS myelination exerts on neurite outgrowth, thus instating a mature phase of restricted structural plasticity (Dours-Zimmermann et al., 2009; Giger et al., 2010).

In adulthood, CSPGs interact with oligodendrocyte progenitor cells (OPCs), mature oligodendrocytes, and myelinated axons. Plasticity of myelin sheaths during adulthood has been proposed as an ongoing, activity-dependent process important for learning, through which neural circuit activity is fine-tuned in response to environmental experience (McKenzie et al., 2014; Xiao et al., 2016; Hill et al., 2018; Hughes et al., 2018). CSPGs potently regulate OPC differentiation and oligodendrocyte process outgrowth and myelination and are strongly expressed by OPCs themselves (Ichihara-Tanaka et al., 2006; Trotter et al., 2010; Lau et al., 2012). Several CSPGs, including BCAN and NG2, are key components of the nodes of Ranvier, where they regulate axonal conductance (Huang et al., 2005; Melendez-Vasquez et al., 2005; Dours-Zimmermann et al., 2009; Hunanyan et al., 2010). NG2, a CSPG involved in key brain functions such as instructive guidance cues, synaptic plasticity, regulation of the nodes of Ranvier as well as blood-brain-barrier biology, has long been used as a specific OPC marker (Butt et al., 1999; Yang et al., 2006; Kucharova and Stallcup, 2010, 2015; Ferrara et al., 2016; Serwanski et al., 2017). BCAN is secreted by OPCs during active myelination, consistent with its involvement in regulating this process (Bekku and Oohashi, 2010).

Despite their potential broad implications in the regulation of axonal functions, the existence of axonal coats in the adult human brain remains unconfirmed. We tested the hypothesis that BCAN and NG2 form axonal coats, i.e., ECM structures ensheathing axons in the healthy adult human brain. To this end, we focused on the MD, a brain region containing abundant axons, easily identifiable within its pars fasciculata, where they are arranged in large bundles.

## Materials and Methods

### Human Subjects

Tissue blocks containing the whole thalamus from healthy control donors were used for these investigations (*n* = 5 for dual immunofluorescence studies; *n* = 3 for electron microscopy studies) (Table 1). All tissue blocks were obtained from the Harvard Brain Tissue Resource Center (HBTRC), NeuroBioBank site, McLean Hospital, Belmont, MA, United States. Neuropathological assessment of each donor did not show diagnostic findings. The cohort did not include subjects with evidence for gross and/or macroscopic brain changes, or clinical history, consistent with cerebrovascular accidents or other neurological disorders. Subjects with Braak and Braak stages III or higher were not included. Review of extensive clinical records and family questionnaires by HBTRC clinicians ruled out psychiatric disorders. None of the subjects had significant history of substance dependence within 10 or more years from death, as further corroborated by negative toxicology reports. Absence of recent substance abuse is typical for samples from the HBTRC, which receives exclusively community-based brain tissue donations.

**TABLE 1:** Sample demographic and descriptive characteristics of the cohort used for immunohistochemical investigations.

Samples for Immunofluorescence Studies					
Case/age/sex	Cause of death/Inflammation	Brain weight (g)	PMI (hrs)	Hemisphere	Time of Death
93/70/F	Myocardial infarction (A, N)	1245	18.0	R	07:29
05/26/M	Unknown	1250	18.3	R	NA
25/53/F	Cancer (C, N)	1330	24.0	R	08:32
20/74/M	Cardiac Arrest (A, N)	1490	15.8	R	08:41
40/74/F	Cardiac Arrest (A, N)	1100	23.0	L	11:30
Total/mean ± SD 59.4 ± 20.6/3F, 2M		1283 ± 142.3	19.8 ± 3.5	1L/4R	

**Samples for Electron Microscopy Studies**					
**Case/age/sex**	**Cause of death/Inflammation**	**Brain weight (g)**	**PMI (hrs)**	**Hemisphere**	**Time of Death**

38/95/F	Myocardial infarction (A, N)	1350	07.1	R	14:50
20/74/M	Cardiac Arrest (A, N)	1490	15.8	R	08:41
92/61/M	Cardiac Arrest (A, N)	1100	10.1	R	12:30
Total/mean ± SD 76.7 ± 17.2/1F, 2M		1313 ± 197.5	11.0 ± 4.4	3R	

*A, acute, no prolonged agonal period; C, chronic, with agonal period; I, infection/inflammatory condition present at time of death; N, no significant infection/inflammation present at time of death.*

### Tissue Processing

Tissue blocks containing the thalamus were dissected from fresh brains and post-fixed in 0.1 M phosphate buffer (PB) containing 4% paraformaldehyde and 0.1 M Na azide at 4°C for 3 weeks, then cryoprotected at 4°C for 3 weeks (30% glycerol, 30% ethylene glycol, and 0.1% Na azide in 0.1 M PB), embedded in agar, and pre-sliced in 2 mm coronal slabs using an Antithetic Tissue Slicer (Stereological Research Lab., Aarhus, Denmark). Each slab was exhaustively sectioned using a freezing microtome (American Optical 860, Buffalo, NY, United States). Sections were stored in cryoprotectant at –20°C. Using systematic random sampling criteria, sections through the thalamus were serially distributed in 26 compartments (40 μm thick sections; 1.04 mm section separation within each compartment). All sections within one compartment/subject were selected for each marker, thus respecting the “equal opportunity” rule (Coggeshall and Lekan, 1996; Gundersen et al., 1999).

#### Primary Antibodies

*NG2* – rabbit polyclonal IgG anti-CSPG4 (55027-1-AP, lot#09000034, Protein Tech Group Inc., Rosemont, IL, United States) raised against a synthetic peptide corresponding to human CSPG4/NG2, GenBank accession # NM_001897.

*Brevican* – rabbit polyclonal IgG anti-brevican (ab106615, lot#GR163248-6, Abcam, Cambridge, MA, United States), affinity purified, raised against a synthetic peptide corresponding to amino acids 539–588 (PTETLPTPRE RNLASPSPST LVEAREVGEA TGGPELSGVP RGESEETGSS) of Human Brevican (NP_940819).

*SMI-312* – mouse monoclonal IgG1 anti-SMI312 (ab24574, lot# B226113), Abcam, Cambridge, MA, United States) recognizing pan-neuronal neurofilaments commonly used as a marker for axons (Ulfig et al., 1998).

### Dual Antigen Immunofluorescence

Antigen retrieval was carried out by placing free-floating sections in Vector Antigen Unmasking solution (1:100 in 0.1 M PB; Vector Labs, Burlingame, CA, United States) heated to 80 degrees °C for 1 h. For dual labeling, sections were co-incubated in primary antibodies (NG2, 2 μl:1000 μl, BCAN, 2 μl:1000 μl; SMI-312, 0.5 μl:1000 μl) in 2% bovine serum albumin (BSA) for 72 h at 4°C. This step was followed by 4 h incubation at room temperature in Alexa Fluor goat anti-mouse 594 (1:300 μl; A-11005, Invitrogen, Grand Island, NY, United States) and donkey anti-rabbit 488 (1:300 μl; A-21206, Invitrogen, Grand Island, NY, United States), followed by 10 min in 1 mM CuSO4 solution (pH 5.0) to block endogenous lipofuscin autofluorescence (Schnell et al., 1999). Sections were mounted and coverslipped using Dako mounting media (S3023, Dako, North America, Carpinteria, CA, United States).

### Electron Microscopy: Immunohistochemistry and Tissue Processing

Antigen retrieval was carried out by placing free-floating sections in Vector Antigen Unmasking solution (1:100 in 0.1 M PB; Vector Labs, Burlingame, CA, United States) heated to 80 degrees °C for 1 h. Sections were then rinsed and incubated in 0.05 M glycine (4°C, 1 h, Sigma Millipore) to bind free aldehydes, followed by rinses and blocking in preblocking solution, which contained 10% normal serum of the secondary antibody host animal, Triton-X100 (0.025%, Roche Applied Science; EM), and cold-water fish gelatin (0.1%, Aurion; EM) for stabilization of ultrastructure. Sections were then incubated in primary antibody (NG2, 2 μl:1000 μl or BCAN, 2 μl:1000 μl) for 72 h at 4°C. For control sections, primary antibodies were omitted. Incubation was enhanced by microwaving (2 min at 150 W, 4°C). After primary antibody incubation, sections were incubated in a gold-conjugated secondary antibody (1:50; UltraSmall ImmunoGold F(ab) fragment of goat anti-mouse IgG; catalog #800.266, Aurion; [RRID:AB_2315632]) in a buffer solution (10% normal goat serum, 10% BSA, 0.2% BSA-c, 0.025% Triton X-100 [Roche Applied Science], and 0.1% cold water fish gelatin [Aurion] in 0.1 M PB), followed by silver enhancement of gold particles (90 min, R-GENT SE-EM kit 500.033, Electron Microscopy Sciences, catalog #255213), quenching with 0.1 M PB rinses, and incubation in enhancement solution (Enhancement conditioning solution 10 × 500.055, Electron Microscopy Sciences, catalog #25830). Sections were photographed using a temporary 0.1 M PB mount on an unsubbed glass slide before the remainder of EM processing. We performed microwave postfixation (6% glutaraldehyde, 2% PFA in 0.1 M PB, 150 W, 15°C) until sample temperatures reached 30–35°C, then left the sections in the fixative to come to room temperature for 30 min, which was followed by 0.1 M PB rinses.

Sections were processed for EM using a protocol optimized for scanning/transmission EM and high-throughput block-face imaging, as described previously (Garcia-Cabezas et al., 2016; Zikopoulos et al., 2018; Liu et al., 2020). Briefly, we postfixed tissue sections for 36 min in 2% osmium tetroxide (Electron Microscopy Sciences) with 1.5% potassium ferrocyanide in 0.1 M PB under vacuum with an initial microwave session (100 W at 4°C, 6 min). After three dH_2_O rinses, we incubated the tissue sections for 30 min in 1% thiocarbohydrazide in dH_2_O (Sigma Millipore), followed by another three dH_2_O rinses. We then incubated tissue sections in a second osmium solution (2% osmium tetroxide in dH_2_O), under vacuum, with an initial microwave session (100 W at 4°C, 6 min), for a total of 36 min. Sections were rinsed in three dH_2_O rinses and stained overnight at 4°C in 1% uranyl acetate (Electron Microscopy Sciences) in dH_2_O. The next day, we rinsed the sections with dH_2_O 3 times, and then incubated them in lead aspartate solution (0.066 g lead nitrate, Electron Microscopy Sciences, in 10 ml of 0.4% L-aspartic acid in dH_2_O, titrated to pH 5.5 using 20% potassium hydroxide solution, Sigma Millipore) for 30 min at 60°C. We then dehydrated the sections in ascending graded ethanol solutions (50, 75, 85, 95, and 100%, 3 min × 5 min each). Sections were then infiltrated with propylene oxide (2 min × 10 min, Electron Microscopy Sciences), followed by a 1:1 mixture of LX112 resin (LX112 Embedding Kits, Ladd Research Industries) and propylene oxide for 1 h, and finally with a 2:1 mixture of LX112 and propylene oxide at 25°C overnight. The following day, the sections were infiltrated with pure LX112 resin for 4 h under vacuum, and then flat embedded in LX112 resin between sheets of Aclar (Ted Pella), and cured for at least 48 h at 60°C. Using a stereoscope, we dissected small cubes of Aclar-embedded tissue, using the photographs of each section taken before EM processing to identify fiduciary landmarks (e.g., blood vessels) and precisely locate our regions of interest, and prepared LX112 resin blocks. These blocks were then cured for ≥48 h at 60°C. For block-face imaging, aluminum pins containing cubes of tissue were prepared using conductive epoxy glue (Chemtronics, catalog #CW2400). We used an ultramicrotome (Ultracut UCT, Leica Microsystems) to expose the surface of the tissue in resin blocks and pins. After exposing the tissue, pins for block-imaging were painted with conductive silver paint (Ted Pella, catalog #16035), which reduces charging artifacts in the scanning electron microscope (SEM). After the tissue was exposed on the resin blocks for the TEM, we cut ∼50 nm ultrathin sections and collected them sequentially on pioloform-coated copper slot grids to form a series of 20–300 sections.

### Data Collection

#### Dual-Immunofluorescence Confocal Microscopy Quantification

A Zeiss Axio Imager M2 with a Lumencor SOLA LED lamp interfaced with StereoInvestigator 10.0 (Microbrightfield Inc., Williston, VT, United States) was used for analysis. The borders of the MD thalamus were delineated using a 1.6× objective according to cytoarchitectonic and myeloarchitectonic criteria as described by Hirai and Jones (1989). Adjacent Nissl and luxol blue stained sections were used as references for delineation of each immunostained section. One set of 40 μm thick serial sections per subject representing the rostral to caudal extent of the MD thalamus (10–12 sections/subject; 1.04 mm section separation within each set) was used for stereology-based quantification. A sampling grid was randomly placed over the MD on each section using the Stereo-Investigator optical fractionator sampling method in order to obtain counting frame sampling sites in a systematic random sampling manner (Gundersen et al., 1999; Dorph-Petersen et al., 2000).

A pilot study was used to determine the optimal grid and counting frame size. On the basis of this pilot study, we chose a grid size of 800 μm × 800 μm and a counting frame size of 300 μm × 300 μm. Using a 63× oil immersion objective (Zeiss Plan Apochromat No. 440760; numeric aperture 1.4; working distance 0.19 mm), we counted all cross-sectional SMI-312-immunoreactive (IR) axons with or without axonal coats, and all NG2- or BCAN-IR axonal coats within each counting frame. Confocal images obtained using a Leica TCS-SP8 confocal microscope with a 100× oil immersion objective were used for confirmation and for measurements of axonal diameter and axonal coat thickness. High-resolution confocal images were scanned at 1 μm intervals through the extent of the *z*-axis, resulting in 26–32 scans per image.

#### Electron Microscopy

Electron microscopy (EM) images were obtained using an 80 kV transmission electron microscope (TEM, 100CX, JEOL) at 2000–26,000×. ROIs were sampled systematically, and images were captured for analysis using a digital camera (DigitalMicrograph, Gatan). EM stacks were aligned manually in Reconstruct (Fiala, 2005). For block-face imaging, pins were mounted into the 3View 2XP System (Gatan) coupled to a 1.5 kV scanning electron microscope (GeminiSEM 300, Carl Zeiss). The surface of the pin was imaged using a backscatter detector at 6.5 nm/pixel resolution. A built-in automated microtome then cut a 50 nm section from the surface of the pin, and the ROIs were imaged again. This way, long series of ≥300 sections were imaged in sequence at each ROI. For series obtained using block-face imaging, we used an algorithm for alignment (GMS3.0, Gatan). EM images and stacks were imported into Reconstruct (Fiala, 2005), where we exhaustively outlined all myelinated axons, identified the distribution of labeling in distinct compartments of the axon, and estimated the major diameter of each axon. Some labeled axons were followed in very long series and reconstructed into 3D models using Reconstruct and Studio Max (Autodesk) for basic smoothing.

## Results

### NG2 and Brevican Form Tubular Structures Surrounding Axons in the Human Mediodorsal Thalamus

**FIGURE 1:**
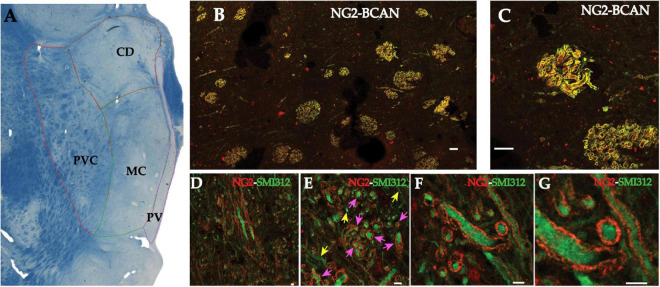
NG2 and BCAN form coats surrounding axons in the human MD thalamus. Photomicrograph of a luxol blue stained section depicting the subregions of the human mediodorsal nucleus sampled for quantification of axonal coats **(A)**. Myelinated fiber bundles were sampled from the parvocellular (PVC), the magnocellular (MC), caudodorsalis (CD) regions of the MD. **(B)** Low magnification (10×) confocal image of NG2 labeling (red) and BCAN labeling (green) in the mediodorsal nucleus of the human thalamus. Labeling for both CSPGs was observed in structures resembling myelinated fiber bundles. Higher magnification images **(C)** revealed that these CSPGs labeled tube-like structures apparently surrounding openings that were consistent with the diameters of single axons. Scale bar equals 30 μm. Confocal micrographs of dual immunofluorescence labeling revealed that these CSPG structures resembling axonal coats, labeled with NG2 (red), surrounded SMI-312 immunoreactive axons (green). **(D)** Low magnification (10× objective) image showing NG2 axonal coats around SMI-312 axons in transverse and cross-section slices of the axons. **(E)** An intermediate magnification image depicting an example of cross-sectional SMI-312 axons surrounded by NG2 coats. Pink arrows indicate axons surrounded by NG2 coats, yellow arrows indicate axons without NG2 coats. **(F)** High-resolution imaging allowed for measurements of the diameter of these axons (3.95 and 3.99 μm) as well as the thickness of the NG2 axonal coats [892 and 856 nanometers; **(G)**]. Scale bars equal 4 μm for **D–G**.

Multiplex immunocytochemistry combined with high-resolution confocal microscopy was used to assess the relationship between SMI-312-IR axons and NG2- and BCAN-IR in the human MD thalamus. NG2- and BCAN-IR were concentrated within axon bundles in the MD (Figure 1A). Within these bundles, NG2- and BCAN-IR were found to co-localize within tube-like structures, resembling myelin sheaths (Figure 1). Dual-labeling with the axonal marker SMI-312 shows that BCAN/NG2-IR tubular structures tightly surround axons (Figure 1). They are referred to here as ‘axonal coats.’ High-resolution confocal microscopy measurements show that the diameter of axonal coats within the human MD is 3.95–3.99 μm and the thickness of their walls is 856–892 nm (Figures 1E,F).

### Axons Within the Human Mediodorsal Thalamic Nucleus Are Predominantly Associated With Axonal Coats

**FIGURE 2:**
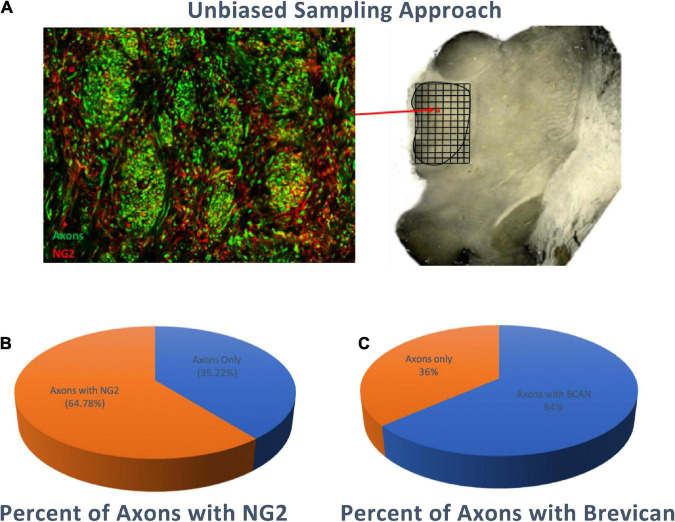
Percentage of Axons surrounded by NG2 or BCAN Coats. Stereology-based sampling was used to quantify the number of SMI-312 immunoreactive axons and axonal coats within bundles of the MD **(A)** approximately 65% (standard deviation ±3.3%) of axons were associated with NG2 immunoreactive coats **(B)** and 64% (standard deviation ±2.7%) were associated with BCAN immunoreactive coats **(C)**.

Unbiased stereology-based sampling in 5 control subjects was used to quantify the percentage of axons in the human MD surrounded by NG2-IR and BCAN-IR axonal coats. Our results show that 64.78% (standard deviation ±3.3%) of axons were surrounded by NG2-IR coats and 64% (standard deviation ±2.7%) by BCAN-IR coats, consistent with predominant colocalization of these CSPGs. Conversely, 35.22% (standard deviation ±3.3%) and 36% (standard deviation ±2.7%) of axons were not associated with NG2-IR and BCAN-IR axonal coats, respectively (Figure 2).

### Ultrastructural Characteristics of NG2- and Brevican-Immunoreactive Axonal Coats

Electron microscopy analysis of BCAN- and NG2-IR axonal coats in the human MD was carried out on a total of 24,148 cross-sections of axon segments from randomly selected sections. We focused on the location of each CSPG with respect to the axon and its myelin sheath and measured the incidence BCAN- and NG2-IR within the axon cytoplasm, just below the myelin sheath outside the axoplasm, or above the myelin sheath on the surface of the myelinated axon.

#### Distribution of Axonal Coat Labeling With Respect to Axonal Segments

In randomly selected photomicrographs of sections labeled for BCAN, 17% of the axon segments showed BCAN-IR interleaved between myelin layers or associated with axon membrane, 10% of axon segments had BCAN-IR on the surface of the myelin sheath, and 73% of axon segments had no BCAN expression. In sections immunolabeled for NG2, 15% of axon segments showed NG2-IR interleaved with the myelin layers or associated with the axon membrane, 6% of axon segments had NG2-IR on the surface of the myelin sheath, and 79% of axon segments had no NG2 expression. Note that discrepancies between confocal and electron microscopy regarding the percentages of axons associated with BCAN-IR and NG2-IR are explained by the fact that EM detects axons with diameters below 0.1–0.2 μm, which are below the resolution of optical microscopy. Studies from our group and others have reported that EM detects 30–50% more myelinated axons compared to confocal microscopy (Zikopoulos and Barbas, 2010; Liewald et al., 2014; Wegiel et al., 2018; Zikopoulos et al., 2018).

#### Axonal Coats Are Associated With Medium- and Large-Size Axonal Coats

**FIGURE 3:**
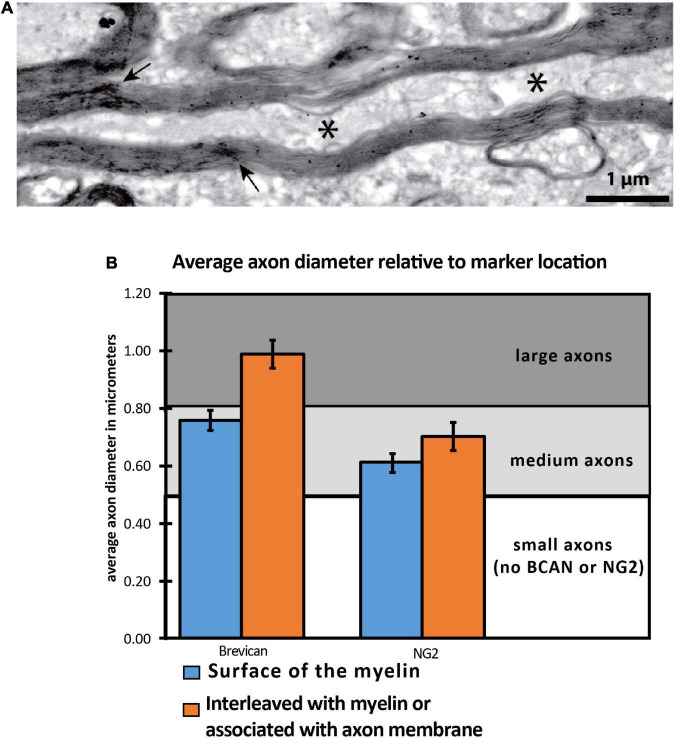
NG2 and BCAN labeling is associated with larger axons. **(A)** Electron microscopy image depicting a cross-sectional axon with myelin labeling and interweaved immunoreactivity for NG2. **(B)** Two-dimensional quantitative analysis of 24,000 axon segments revealed that BCAN and NG2 labeling within the cytoplasm was more frequently observed in larger axons. The * symbol indicates location of the axon.

We assessed whether the relationship between BCAN- and NG2-IR axonal coats and myelin sheaths varies with axon size (Figure 3). BCAN-IR axonal coats were not detectable in myelinated axons with a diameter below 0.63 μm on average. In contrast, medium-size axons (average diameter of 0.76 μm), showed a layer of BCAN-IR surrounding the myelin sheaths. Larger axons, with an average diameter of 0.9907 μm, predominantly showed BCAN-IR interleaved with myelin lamellae or associated with the axon membrane. A similar pattern was observed for NG2-IR axonal coats. Small myelinated axons, with a diameter of 0.53 μm or smaller, did not display NG2-IR axonal coats. Axons with an average diameter of 0.61 μm, showed NG2-IR surrounding the myelin sheets. In larger axons, with an average diameter 0.70 μm, NG2-IR was interleaved with the myelin sheaths or associated with the axon membrane. These findings indicate that BCAN- and NG2-IR axonal coats are predominantly associated with medium- and large-size axons, and that their spatial relationship with the axon and its myelin sheets varies according to the axon size.

#### Spatial Relationships Between Axonal Coats, Axons, and Myelin

**FIGURE 4:**
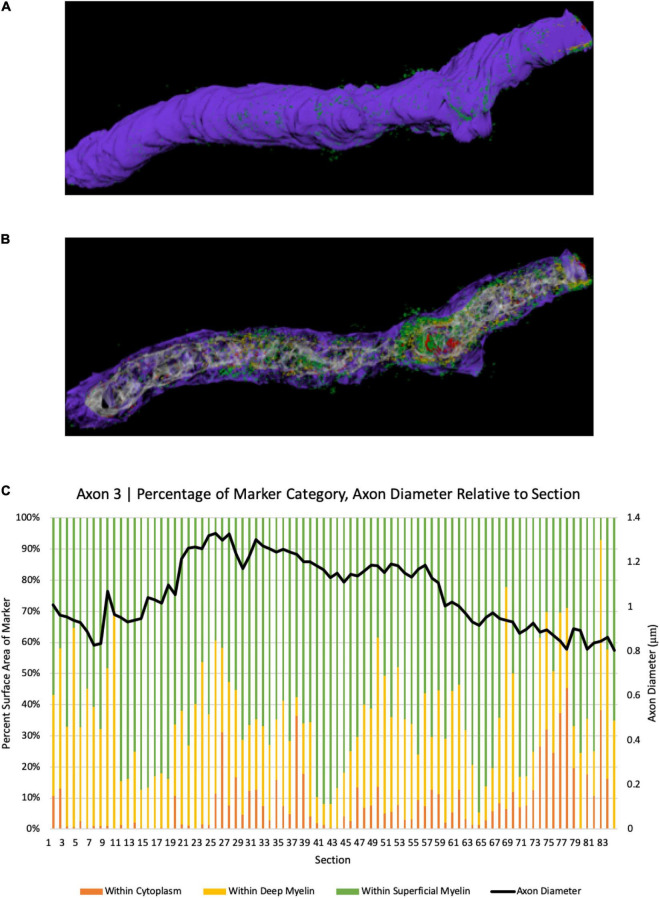
3D reconstruction of BCAN labeling in a single axon segment. **(A)** Single axonal segment (Axon 3) 11.63 μm long, depicts the outside aspect of the axon. Reconstruction shows the same axon, electronically transected along its longitudinal axis to show the distribution of BCAN in the cytoplasm (red), beneath and between the myelin sheaths **(B)**. **(C)** Quantitative analysis of axon diameter and marker category percentage for each section. *Y*-axes depict percent surface area of BCAN labeling in each axonal compartment category (left); legend: Purple, myelin; white-gray, cytoplasm; red, within cytoplasm; yellow, marker within deep myelin; green, marker within superficial myelin, and the corresponding axon diameter of each section in microns (right), for each transected axon section analyzed as indicated on the *x*-axis.

To further explore the spatial relationship between axonal coats and myelinated axons, we analyzed 231 axon segments whose length could be studied across at least 300 nanometers. BCAN-IR was detected within the cytoplasm in 38% of the time, between the myelin lamellae 33% of the time, and on the surface of the myelin sheaths 29% of the time (Figures 3, 4). We then examined longer segments (>10 μm) of four axons in 3D to show the pattern of BCAN-IR with respect to myelinated axons. To do this, we reconstructed each axon electronically ‘transected’ longitudinally to show the location of BCAN-IR (Figure 4). The results show a regular pattern of BCAN-IR, weaving from the surface of the myelin coating to the inside of the axon with a period of approximately 2 μm.

## Discussion

In the human MD, our results show that the CSPGs BCAN and NG2 form tubular sheaths enveloping large- and medium-sized myelinated axons. The term ‘axonal coats,’ originally suggested by Bruckner et al. (2008) and Morawski et al. (2010), aptly describes these ECM/CSPG structures. We describe, to our knowledge for the first time, the ultrastructure of axonal coats, showing that they associate predominantly with medium to large size axons, forming complex structures in relation to myelin sheaths. These findings add to existing evidence for structural relationships between the ECM and neural axons and further point to the involvement of CSPGs in axonal functions. We show that BCAN and NG2 are present not only in the axon initial segment and nodes of Ranvier, where they are thought to regulate neuronal excitability and saltatory conduction, respectively (Butt et al., 1999; John et al., 2006; Bekku et al., 2009; Giger et al., 2010; Hunanyan et al., 2010), but they also surround the axons and interleave with myelin sheaths. In the context of growing support for CSPG functions in the regulation of axon guidance, fasciculation, and myelination, our findings provide evidence for axonal coats as an ECM structure potentially underlying these functions. Speculatively, given the dynamic role of myelination in the adult brain, where rapid activity-dependent changes in axon myelination support neural plasticity, we put forth the hypothesis that axonal coats may serve to stabilize myelin sheaths in a manner analogous to the role played by PNNs and perisynaptic ECM aggregates in synaptic regulation.

### Brevican- and NG2-Immunoreactive Axonal Coats: Association With Medium and Large Axons

NG2- and BCAN-IR axonal coats within the human MD were primarily associated with medium and large axons (Figure 3). This relationship suggests that axonal coats containing these CSPGs may contribute to the high conductance velocity and spike frequencies typical of larger axons, which are mostly involved in long-range pathways (Arbuthnott et al., 1980; Friede et al., 1984; Perge et al., 2009, 2012; Horowitz et al., 2015). Well-established CSPG functions such as regulating ionic homeostasis and providing structural support offer further evidence for this possibility. Interestingly, the predominant association of axonal coats with medium and large axons parallels the reported OPC preference to repair minor myelin damage in larger axons (Snaidero et al., 2020; Call and Bergles, 2021).

NG2 is a transmembrane CSPG selectively expressed in the brain OPCs and pericytes (Levine and Card, 1987; Stallcup and Beasley, 1987; Levine et al., 1993; Karram et al., 2008; Moransard et al., 2011; Huang et al., 2014; Stallcup, 2018; Li et al., 2020; Melrose et al., 2021; Chelyshev et al., 2022). OPCs are a major source of mature oligodendrocytes in the adult brain – however, these latter cells are not known to express NG2. Thus, although our study cannot exclude it, it is not likely that the NG2-IR detected in axonal coats is simply a component of the myelin sheaths.

Brevican is secreted into the ECM, where it plays a variety of functional roles, from entering in the composition of PNNs to regulating neuritic functions. The source of BCAN contributing to axonal coats is more difficult to infer, as this CSPG is expressed by neuronal and glial cells, including OPCs (Seidenbecher et al., 2002; John et al., 2006). While BCAN is not expressed by fully mature oligodendrocytes (Ogawa et al., 2001), it is secreted by OPCs during active myelination, consistent with its involvement in regulating this process (Bekku and Oohashi, 2010). Thus, OPCs are also good candidates as a source of BCAN for axonal coats. Notably, evidence supports a key role for BCAN in bridging between the intracellular neuronal domain and the ECM, as it attaches to the surface of the neuronal membrane and interacts with the hyaluronan acid-based extracellular domain (Seidenbecher et al., 2002). This property may be mediated by the adhesion molecule neurofascin 186 kDa isoform (NF-186), which directly links the intracellular cytoskeleton to BCAN-based ECM (Desmazieres et al., 2014). Indeed, interactions between NF-186 and brevican have been found to stabilize the axon initial segment and node of Ranvier (Hedstrom et al., 2007; Kriebel et al., 2012). Speculatively, these observations may account for the axonal coat pattern observed at the ultrastructural level, as BCAN-IR was found to interleave with myelin lamellae as well as associated with the axon.

### Ultrastructure of NG2- and Brevican-Axonal Coats

Our ultrastructural analyses showed an intriguing BCAN- and NG2-IR distribution pattern, with a regular wave-like pattern moving from the surface of the myelin sheath, through the myelin layers, within the axon and back to the surface of the myelin sheath (Figure 4). This pattern was more frequently associated with larger axons, suggesting that its functional significance may perhaps be related to structural support. The interleaving pattern formed by axonal coats and myelin lamellae showed a striking regularity, with a period of 2 μm, suggesting a tightly regulated geometric relationship between these structures (Figure 4). Such a short, 2 μm, period is not compatible with the possibility that sites where the axonal coats associate with the axon may correspond to the nodes of Ranvier. This is because the distance between two nodes is expected to be greater, reported to be between 27 and 154 μm at least in the mouse cortex (Chong et al., 2012; Tomassy et al., 2014; Arancibia-Carcamo et al., 2017). Furthermore, the myelin sheath is clearly visible above the sites where BCAN- and NG2-IR are within or on the surface of the axonal membrane (Figure 4). We put forward the hypothesis that, in larger axons, the interleaving pattern formed by axonal coats and myelin sheaths may help to anchor and stabilize these latter with respect to the axonal cytoskeleton. Speculatively, this function may be analogous to the role played by PNNs and perisynaptic ECM aggregates around active synapses (Faissner et al., 2010; Frischknecht et al., 2014; Ferrer-Ferrer and Dityatev, 2018). Growing evidence for a dynamic, activity-dependent role of myelination, shown to represent a critical aspect of plasticity, is consistent with this hypothesis (McKenzie et al., 2014; Xiao et al., 2016; Hill et al., 2018; Hughes et al., 2018).

### Axonal Plasticity and Axonal Conductance

Myelin sheath plasticity occurs throughout adulthood to fine-tune neural circuit activity in response to environmental experience (McKenzie et al., 2014; Xiao et al., 2016; Hill et al., 2018; Hughes et al., 2018). Several lines of evidence suggest that axonal coats may contribute to this process. During late development, CSPGs are key contributors to the powerful inhibition that CNS myelination exerts on neurite outgrowth, thus instating a mature phase of restricted structural plasticity (Dours-Zimmermann et al., 2009; Giger et al., 2010). Furthermore, several studies in rodents suggest that NG2-OPCs contribute to axonal plasticity. For example, sensory deprivation, either *via* whisker trimming or ocular deprivation, causes changes in the number and distribution of NG2-OPCs in these regions, and is associated with altered axonal conductance (Mangin et al., 2012; Etxeberria et al., 2016).

Several CSPGs, including BCAN and NG2, are key components of the nodes of Ranvier. In particular, BCAN has been shown to play a role in determining the specialization and composition of the ECM nodal matrix, particularly in large diameter axons (Bekku et al., 2009). It is tempting to speculate that there may be structural and functional relationships between BCAN/NG2-IR axonal coats and these CSPGs within the nodes of Ranvier. Although it is beyond the scope of the present study, future investigations may assess such relationships and the potential continuity between axonal coats and peri-nodal ECM.

### Axon Fasciculation

In the developing brain, CSPGs have been proposed to form axon guidance pathways for thalamocortical axons (Bicknese et al., 1994; Anderson et al., 1998). This function, related to the CSPG inhibitory properties, has been proposed to be a key aspect of axonal fasciculation (Bicknese et al., 1994; Snow et al., 2003). In particular, NG2 and BCAN were reported to have inhibitory effects on axonal growth (Davies et al., 2004; Tan et al., 2005), supporting the hypothesis that BCAN- and NG2-IR axonal coats may contribute to axon fasciculation. This possibility may be particularly relevant in the context of our study, which was focused on large axon bundles within the MD. Our findings suggest that the large percentage of axonal coats detected in myelinated fiber bundles within the MD may be related to their role in axon fasciculation, promoting the adherence of axons into segregated fiber bundles. It is possible that the high density of large, myelinated fiber bundles in the MD, with a substantial representation of axonal coats, may account for discrepancies between our findings and those reported in the human lateral geniculate nucleus by Lendvai et al. (2012).

### Implications for Brain Disorders

Several lines of evidence point to a CSPG dysregulation in a growing number of brain disorders. We previously identified abnormal CSPG expression in the amygdala, entorhinal cortex, prefrontal cortex, and thalamic reticular nucleus of subjects with schizophrenia (Pantazopoulos et al., 2010, 2015; Mauney et al., 2013; Steullet et al., 2018). Our group has also reported widespread ECM abnormalities involving genes encoding for CSPGs, matrix metalloproteases, link proteins, and semaphorins in several cortical and subcortical brain regions (Pantazopoulos et al., 2021). Recent genetic studies, including GWAS, have reported associations of genetic polymorphisms for genes encoding specific CSPGs, such as neurocan, neuroglycan-C, and PTPRZ1, and molecules involved in the regulation of CSPGs including matrix metalloproteases (Buxbaum et al., 2008; Dow et al., 2011; Ripke and Consortium, 2011; Bespalova et al., 2012; Muhleisen et al., 2012; McGrath et al., 2013; Consortium, 2014; Ripke and Schizophrenia Working Group of the Psychiatric Genomics, 2014), suggesting that CSPG abnormalities represent core aspects of the neuropathophysiology of schizophrenia. NG2 has been implicated in this disorder in at least one study (de Vrij et al., 2019).

Extensive evidence from functional imaging studies has identified disrupted cortico-thalamic functional connectivity in subjects with schizophrenia (Schlosser et al., 2003a,b; Marenco et al., 2012; Anticevic et al., 2014; Saalmann, 2014; Canu et al., 2015; Cho et al., 2015; Hoflich et al., 2015; Lui et al., 2015). Impaired connectivity of this pathway is believed to impact several clinical aspects of this disorder, including psychosis, attention sensory motor integration, and emotional processing. DTI/fiber tractography studies in subjects with schizophrenia provide strong evidence for a disruption of anatomical connectivity and white matter integrity between cortical areas and thalamus (Rose et al., 2006; Kim et al., 2008; Kito et al., 2009; Oh et al., 2009; Antonius et al., 2011; Sui et al., 2011; Kubota et al., 2012; Marenco et al., 2012). Furthermore, evidence for myelination deficits comes from reports of altered expression of myelin-related proteins and modest oligodendrocyte reduction in the MD and other thalamic nuclei (Schmitt et al., 2004; Byne et al., 2006, 2008; Beasley et al., 2009; Martins-de-Souza et al., 2010). In this context, deficits in axonal coats composed of CSPGs from OPCs may contribute to thalamo-cortical dysconnectivity in schizophrenia.

White matter and oligodendrocyte pathology have also been reported in Alzheimer’s disease and proposed to precede disease symptoms (Lee et al., 2016, 2018; Araque Caballero et al., 2018; Nasrabady et al., 2018). Recent studies support a role for CSPG abnormalities in Alzheimer’s disease, including PNN deficits, CSPG expression in amyloid beta plaques, decreased CSF levels of NG2, and increased levels of chondroitin-4-sulfate (Bruckner et al., 1999; Baig et al., 2005; Morawski et al., 2010; Lendvai et al., 2013; Nielsen et al., 2013, 2014; Vegh et al., 2014; Howell et al., 2015; Yang et al., 2017; Cattaud et al., 2018; Schultz et al., 2018). Potential decreases of axonal coats may therefore contribute to white matter connectivity deficits in these diseases.

## Conclusion

In summary, our data support the existence of a novel ECM structure, axonal coats, in the human MD thalamus, composed of the CSPGs NG2 and BCAN, interweaved between myelin sheaths and axonal plasma membranes. CSPG axonal coats may contribute to several aspects of axon regulation, including fasciculation, axonal guidance, and stabilization of myelin sheath. CSPG involvement in several brain disorders, including schizophrenia and Alzheimer’s disease, raises the possibility that axonal coat abnormalities may contribute to connectivity dysfunction reported in these disorders.

## Data Availability Statement

The data that support the findings of this study are available from the authors upon reasonable request.

## Ethics Statement

Postmortem human brain samples included in this study were obtained from the Harvard Brain Tissue Resource Center (HBTRC), an NIH NeuroBioBank site. HBTRC protocols for collection and distribution of human brain specimens for research purposes were reviewed and approved by the Mass General Brigham Institutional Review Board. Written informed consent for brain recovery and use for research was provided by the donors’ legal next of kin/legal representative.

## Author Contributions

All authors listed have made a substantial, direct, and intellectual contribution to the, work, and approved it for publication.

## Conflict of Interest

The authors declare that the research was conducted in the absence of any commercial or financial relationships that could be construed as a potential conflict of interest.

## Publisher’s Note

All claims expressed in this article are solely those of the authors and do not necessarily represent those of their affiliated organizations, or those of the publisher, the editors and the reviewers. Any product that may be evaluated in this article, or claim that may be made by its manufacturer, is not guaranteed or endorsed by the publisher.
